# Serum liver enzymes and diabetes from the Rafsanjan cohort study

**DOI:** 10.1186/s12902-022-01042-2

**Published:** 2022-05-12

**Authors:** Mojgan Noroozi Karimabad, Parvin Khalili, Fatemeh Ayoobi, Ali Esmaeili-Nadimi, Carlo La Vecchia, Zahra jamali

**Affiliations:** 1 Molecular Medicuine Research Center, Research Institute of Basic Medical Sciences, Rafsanjan University of Medical Sciences, Rafsanjan, Iran; 2 Department of Epidemiology, School of Public Health, Social Determinants of Health Research Centre, Rafsanjan University of Medical Sciences, Rafsanjan University of Medical Sciences, Rafsanjan, Iran; 3 Occupational Safety and Health Research Center, NICICO, World Safety Organization and Rafsanjan University of Medical Sciences, Rafsanjan, Iran; 4 Non-Communicable Diseases Research Center, Rafsanjan University of Medical Sciences, Rafsanjan, Iran; 5 Department of Clinical Sciences and Community Health, Università Degli Study Di Milano, 20133 Milan, Italy

**Keywords:** Diabetes, Liver enzymes, Alkaline phosphatase, Y-glutamyltransferase, Aspartate amino-transferase, Alanine aminotransferase, Prospective epidemiological research studies in IrAN

## Abstract

**Background:**

We evaluated the relation between ALT, AST, GGT and ALP with diabetes in the Rafsanjan Cohort Study.

**Materials and methods:**

The present study is a cross-sectional research including 9991 adults participated via sampling. We used data obtained from the Rafsanjan Cohort Study (RCS), as a part of the prospective epidemiological research studies in IrAN (PERSIAN). Elevated serum levels of ALT, AST, GGT and ALP were defined according to the reference range of the laboratory in the cohort center. Serum liver enzymes levels within the normal range were categorized into quartiles, and their relationship with diabetes was evaluated by logistic regressions.

**Findings:**

In present study, elevated serum levels of ALT, AST, GGT, and ALP were associated with increased odds of diabetes (adjusted ORs: 1.81, 95%CI 1.51–2.17; 1.75, 95%CI 1.32–2.32; 1.77, 95%CI 1.50–2.08; 1.60, 95%CI 1.35–1.90 respectively). Also, in subjects with normal levels of ALT, GGT and ALP, a dose–response increase was shown for diabetes.

**Conclusion:**

Elevated levels of ALT, AST, GGT and ALP are related to a higher odds of diabetes. Also, increased levels of ALT, GGT and ALP even within normal range were independently related with the increased odds of diabetes. These results indicated the potential of elevated liver enzymes as biomarkers for the possible presence of diabetes.

**Supplementary Information:**

The online version contains supplementary material available at 10.1186/s12902-022-01042-2.

## Introduction

The liver has an essential part in the maintenance of glucose homeostasis [1]. A number of markers indicating liver injury, including γ-glutamyl-transferase (GGT), aspartate aminotransferase (AST), Alkaline phosphatase (ALP) and alanine aminotransferase (ALT) are measures for non-alcoholic fatty liver disease (NAFLD) which has been associated with insulin resistance [2] and the risk of diabetes [3].

Diabetes mellitus is one of the key public health problem as well as a leading factor of mortality and morbidity globally [4, 5]. As stated by International Diabetes Federation (IDF), approximately 1 out of 11 adult people in the world would be afflicted with diabetes mellitus [6]. Nearly 80% of diabetic subjects are residents of low and middle-income countries, and countries from South-East Asia especially are influenced by this disease [6]. Diabetes has been related to various liver illnesses such as NAFLD, hepatocellular carcinoma and cirrhosis [3, 7, 8].

These liver diseases are regarded as major contributors to death among diabetic patients [9]. An important marker of liver damage in NAFLD disease is altered liver enzyme levels [10] which are biological markers linking liver disease and diabetes [11]. Specific focus has been made on the contribution of liver enzymes to prediction of diabetes. In this respect, although many studies have shown a relation between diabetes and elevated liver enzymes, the results remain inconsistent [12]. Some studies showed significant relationship between high levels of AST, ALT, GGT and diabetes [13, 14]. In another study, a significant increase was observed for GGT, ALT and ALP levels but not AST [11]. Some studies showed that significant increases in ALT and AST are associated with diabetes [7, 15, 16]. On the other hand, in some studies, only an increase in GGT was associated with diabetes [17]. Considering the high prevalence of diabetes in Iran and its implications on cardiovascular diseases [18], it is of interest to determine the relationship between the levels of liver enzymes and diabetes. Our aim was to investigate the correlation of the level of liver enzymes with diabetes in the adult population of Rafsanjan. Moreover, we evaluated the association between liver enzyme levels within their normal ranges and the odds of diabetes.

## Materials and methods

### Research design and selection of patients

The present cross-sectional study was derived from the recruitment phase of Rafsanjan cohort study (RCS) and it was part of the prospective epidemiological researches in in IrAN (PERSIAN) [19]. Briefly, RCS is a cohort population based study that initiated in August 2015 in Rafsanjan, an area in the southeast of Kerman province of Iran. The inclusion criterion of the study has been age between 35 and 70 years from both genders. The enrollment phase ended in December 2017, and a 15-year follow-up is planned. About 14,827 individuals were invited to participate. A total number of 9991 subjects voluntarily participated after signing the written informed consent form [20]. Among this population, 9941 subjects referred for blood sampling. We excluded subjects with missing data of antidiabetic medications. Finally 9895 subjects were considered. There were less than 2% of missing values for all variables and because missing values were completely at random (MCAR), we use complete case analysis to handle missing data. The protocol of research was considered based on Persian Cohort Study [19] and has been confirmed by Ethics Committee of Rafsanjan University of Medical Sciences (Ethics code: ID: IR.RUMS.REC.1399.243).

### Data collection

In this step, structured interview was held with all the participants to complete validated questionnaires including demographic data, socioeconomic status, alcohol consumption, smoking, opium use, disease history, nutrition, and physical activity. Anthropometric measurements were done for all the subjects. Moreover, we measured blood pressure (BP) twice on each arm in millimeter of mercury (mm Hg). Socio-economic condition was also determined using wealth score index (WSI), which was calculated by multiple correspondence analysis (MCA) of economic and social variables [21]. According to WSI, the population under study was divided into four groups: low-class, low-middle class, middle-high class, high class. To assess the intensity of physical activity, metabolic equivalent of task (MET) was used. Physical activity was evaluated according to 24-h physical activities as well as a 22-item questionnaire, which was categorized as low (≤ 35.29 MET-hours per week), moderate (35.30–40.32 MET-hours per week) and heavy (≥ 40.32 MET-hours per week) groups based on the 25th and 75th percentile. Validity of the questionnaires was measured in the PERSIAN Cohort study [19]. The variables such as age, gender, body mass index, physical activity, education, wealth status index, cigarette smoking, alcohol drinking, opium usage, family history of diabetes (yes/no), hypertension (yes/no), hepatitis (yes/no), triglycerides, LDL cholesterol, HDL cholesterol, use of hepatotoxic drugs (yes/no) and fatty liver (yes/no) were considered as covariates. ALT, AST, GGT and ALP were the independent variables. Diabetes was considered as the dependent variables.

### Biochemical measurements

We took the blood samples from 7:00 to 9:00 AM following 12 to 14 h of fasting. We assayed fasting blood glucose (FBG), total cholesterol, low-density lipoprotein (LDL) cholesterol, high-density lipoprotein (HDL) cholesterol,, triglycerides (TG), S.G.O.T (AST), alkaline phosphatase (ALP) and S.G.P.T (ALT) by a biotecnica analyzer (BT 1500, Italy) at central laboratory of the cohort center. Accuracy and precision of all methods were performed in accordance with the relevant guidelines and regulations.

### Definition of terms

Diabetes was described as: FBG ≥ 126 mg/dL or receiving the antidiabetic drugs [22]. Elevated serum ALT, AST, GGT and ALP levels were defined according to the reference range of the laboratory. Elevated serum levels of both ALT and AST were defined > 40 U/L in men and > 35 U/L in female. Elevated serum GGT levels were described as > 54 U/L among males and > 37 U/L among females. Elevated serum ALP levels were defined as > 306 U/L in both genders. The subjects in the normal range were categorized into quartiles. In the normal range, ALT, AST, GGT and ALP levels were divided into the following quartiles: for ALT ≤ 14 U/L, 15–19 U/L, 20–25 U/L, and 26–40 U/L in males; and ≤ 12 U/L, 13–15 U/L, 16–20 U/L, and 21–35 U/L in females; for AST ≤ 16 U/L, 17–19 U/L, 20–23 U/L, and 24–40 U/L in males and ≤ 14 U/L, 15–16 U/L, 17–20 U/L and 21–35 U/L in women. The quartiles for GGT were as follows: ≤ 18 U/L, 19–23 U/L, 24–31 U/L, and 32–54 U/L in men; and ≤ 14 U/L, 15–18 U/L, 19–23 U/L, and 24–37 U/L in women. The quartiles for ALP included ≤ 177 U/L, 178–209 U/L, 210–244 U/L, and 245–306 U/L in both genders.

### Statistical analyses

Frequency (%) for categorical variables and mean (SD: standard deviation) for the quantitative variables were used and baseline characteristics were compared across the groups of our study (non-diabetics, diabetics) and serum concentrations of liver enzymes (elevated, normal**),** using chi-square (χ^2^) and t-test for continuous and categorical variables, respectively.

In addition, we used dichotomous logistics regression analysis to determine the odds ratios (ORs) and the corresponding 95% confidence intervals (CI) for the relation of diabetes to the level of liver enzymes. All models were assessed for multicollinearity. Findings of the model indicated a prominent collinearity between cholesterol and LDL cholesterol, and accordingly, we selected LDL cholesterol in the regression model for further analyses. We used five models in the regression analysis and confounding variables were identified. Potential confounding variables were introduced sequentially into the models. Moreover, the base-line model (crude model) was stratified on the condition of serum concentrations of liver enzymes**.** In addition, the adjusted model 1 included for gender, age, education years and wealth status index and model 2 had further adjustment to the variables that were related with the life style (cigarette smoking, alcohol drinking, opium consumption), BMI (continuous variable) and level of the physical activities (continuous variable). The adjusted model 3 had additional adjustment for hypertension (yes/no), family history of diabetes in first-degree relatives (yes/no) and family history of diabetes in second-degree relatives (yes/no). The adjusted model 4 entails each variable in the adjusted model 3, as well as HDL (continuous variable), triglycerides (continuous variable), LDL (continuous variable), use of hepatotoxic drugs (yes/no) and fatty liver (yes/no).

## Results

**Fig. 1:**
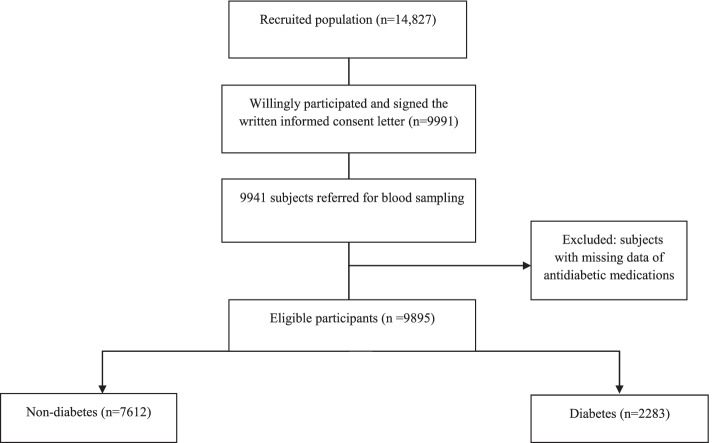
Flowchart of the study design of diabetes in Rafsanjan cohort study

Figure 1 shows the flow chart of the study design of diabetes in Rafsanjan cohort study. A total of 9895 subjects from the base-line phase of Rafsanjan adult Cohort Study were included in the present study. Of these, 4605 (46.54%) were males and 5290 (53.46%) females. The prevalence of diabetes was 23.05%. Among diabetes population (*n* = 2283), about 81.25% (*n* = 1855) had history of diabetes used hypoglycemic agents and 18.75% (*n* = 428) were newly-diagnosed diabetes. The median (interquartile range) of diabetes duration was 4 (2–10) years. Table 1 gives sociodemographic features, personal habits, lifestyle, anthropometric measures, laboratory blood tests as well as clinical risk factors in non-diabetics and diabetics. Educational status was lower in diabetic participants. The frequency of characteristics of diabetic subjects were as follows: 6.36% for alcohol consumption, 21.46% for cigarette smoking, 21.90% for opium consumption, 45.60% for hypertension, 15.46% for fatty liver, 48.53% for use of hepatotoxic drugs. Women were more frequent diabetics than men and the prevalence of diabetes was higher among subjects having lower levels of physical activity than those with higher levels of physical activity and among the subjects with higher levels of BMI. Diabetes prevalence was also higher in subjects with older age and first relative history of diabetes. Elevated AST, ALT, GGT and ALP levels were higher in diabetic subjects compared to non-diabetic subjects (4.38% vs 2.55%, 11.40% vs 8.17%, 16.71% vs 8.26%, and 15.12% vs 8.16%, respectively) (Table 1).

**Table 1:** Demographic, selected medical and laboratory characteristics of study participants (*n* = 9895)

Characteristics	All (*n* = 9895)	Non-Diabetes (*n* = 7612)	Diabetes (*n* = 2283)	*P*-Value
**Age- years. no. (%)**				< 0.001
35–45	3672(37.11)	3367(44.23)	305(13.36)	
46–55	3051(30.83)	2327(30.57)	724(31.71)	
≥ 56	3172(32.06)	1918(25.20)	1254(54.93)	
Mean ± SD	49.92 ± 9.56	48.27 ± 9.24	55.61 ± 8.35	< 0.001
**Gender- no. (%)**				< 0.001
Female	5290(53.46)	3899(51.22)	1391(60.93)	
Male	4605(46.54)	3713(48.78)	892(39.07)	
**Education-no. (%)**				< 0.001
≤ 5 years	3476(35.15)	2344(30.81)	1132(49.58)	
6–12 years	4795(48.48)	3892(51.16)	903(39.55)	
≥ 13 years	1619(16.37)	1371(18.02)	248(10.86)	
**Physical activity -no. (%)**				< 0.001
Low	3834(38.75)	2755(36.19)	1079(47.26)	
Moderate	5034(50.87)	3975(52.22)	1059(46.39)	
Heavy	1027(10.38)	882(11.59)	145(6.35)	
Mean ± SD	36.01 ± 10.00	39.16 ± 6.56	37.56 ± 5.23	< 0.001
**BMI- no. (%)**				< 0.001
< 25	2850(28.81)	2416(31.76)	434(19.01)	
25–29.9	4055(41.00)	3112(40.90)	943(41.31)	
≥ 30	2986(30.19)	2080(27.34)	906(39.68)	
Mean ± SD	27.81 ± 4.92	27.41 ± 4.82	29.19 ± 4.98	< 0.001
**WSI- no. (%)**				< 0.001
Low	2317(23.44)	1720(22.62)	597(26.15)	
Low-middle	2841(28.74)	2113(27.79)	728(31.89)	
Middle-high	3966(40.12)	3122(41.06)	844(36.97)	
High	762(7.71)	648(8.52)	114(4.99)	
**Alcohol consumption- no. (%)**				< 0.001
Yes	986(9.98)	841(11.07)	145(6.36)	
No	8889(90.02)	6755(88.93)	2134(93.64)	
**Cigarette smoking-no. (%)**				< 0.001
Yes	2533(25.65)	2044(26.91)	489(21.46)	
No	7342(74.35)	5552(73.09)	1790(78.54)	
**Opium consumption- no. (%)**				0.030
Yes	2329(23.58)	1830(24.9)	499(21.90)	
No	7546(76.42)	5766(75.91)	1780(78.10)	
**Hypertension- no. (%)**				< 0.001
Yes	2235(22.59)	1194(15.69)	1041(45.60)	
No	7660(77.41)	6418(84.31)	1242(54.40)	
**Family history of diabetes in first-degree relatives—no. (%)**				< 0.001
Yes	4879(49.31)	3432(45.09)	1447(63.38)	
No	5016(50.69)	4180(54.91)	836(36.62)	
**Family history of diabetes in second-degree relatives—no. (%)**				0.971
Yes	2430(24.56)	1870(24.57)	560(24.53)	
No	7465(75.44)	5742(75.43)	1723(75.47)	
**Fatty liver**				< 0.001
Yes	1010(10.21)	657(8.63)	353(15.46)	
No	8885(89.79)	6955(91.37)	1930(84.54)	
**Hepatitis- no. (%)**				0.269
Yes	28(0.28)	24(0.32)	4(0.18)	
No	9867(99.72)	7588(99.68)	2279(99.82)	
**Use of hepatotoxic drugs**				< 0.001
Yes	2382(24.07)	1274(16.74)	1108(48.53)	
No	7513(75.93)	6338(83.26)	1175(51.47)	
**Cholesterol**				
Mean ± SD	198.66 ± 38.07	198.83 ± 36.14	198.11 ± 43.80	0.476
**Triglycerides**				
Mean ± SD	160.84 ± 74.87	160.18 ± 96.02	199.14 ± 145.78	< 0.001
**LDL cholesterol**				
Mean ± SD	108.18 ± 30.31	109.59 ± 28.92	103.47 ± 34.04	< 0.001
**HDL cholesterol**				
Mean ± SD	57.75 ± 10.88	57.95 ± 10.83	57.07 ± 10.98	0.001
**FBS**				
Mean ± SD	113.28 ± 39.09	99.24 ± 9.13	160.25 ± 59.21	< 0.001
**AST—no. (%)**				
Elevated	294(2.97)	194(2.55)	100(4.38)	0.001
Mean ± SD	21.37 ± 9.44	19.55 ± 8.29	20.07 ± 13.43	0.003
**ALT—no. (%)**				
Elevated	882(8.92)	622(8.17)	260(11.40)	< 0.001
Mean ± SD	25.82 ± 20.15	20.91 ± 14.50	23.51 ± 17.20	< 0.001
**GGT- no. (%)**				
Elevated	1010(10.21)	629(8.26)	381(16.71)	< 0.001
Mean ± SD	35.20 ± 44.93	25.93 ± 23.84	32.88 ± 33.68	< 0.001
**ALP- no. (%)**				
Elevated	966(9.76)	621(8.16)	345(15.12)	< 0.001
Mean ± SD	235.02 ± 79.65	220.92 ± 64.96	241.27 ± 69.34	< 0.001

*Abbreviations:* *BMI* body mass index, *WSI* wealth score index, *FBS* Fasting blood sugar, *AST* aspartate aminotransferase, *ALT* alanine aminotransferase, *ALP* alkaline phosphatase, *GGT* γ-glutamyl transferase

Table 2 gives the associations of the liver enzymes with the base-line variables, which are usually related to diabetes. Serum ALT, GGT and ALP were associated to age, education and WSI. Serum ALT and GGT activity were also related to the sex. Additionally, serum AST, GGT and ALT activities were related to fatty liver. All liver enzymes showed a significant positive association with physical activity, BMI, FBS, cholesterol, triglycerides and LDL cholesterol levels. ALT, AST and ALP had a significantly positive relationship with alcohol consumption. GGT and ALP had a significantly positive association with cigarette smoking, whereas ALT and ALP had a significantly positive association with opium consumption.

**Table 2:** Prevalence of elevated serum liver enzymes in study participants (*n* = 9895)

Characteristic	Elevated ALT	*P* -value	Elevated AST	P -value	Elevated GGT	*P* -value	Elevated ALP	*P* -value
**Age cat- no. (%)**		< 0.001		0.905		< 0.001		< 0.001
35–45	438(49.27)		112(37.58)		310(30.45)		215(22.10)	
46–55	268(30.15)		94(31.54)		335(32.91)		344(35.35)	
≥ 56	183(20.58)		92(30.87)		373(36.64)		414(42.55)	
**Gender- no. (%)**		< 0.001		0.621		0.001		0.827
Female	348(39.15)		155(52.01)		595(58.45)		523(53.75)	
Male	541(60.85)		143(47.99)		423(41.55)		450(46.25)	
**Education-no. (%)**		< 0.001		0.090		< 0.001		< 0.001
≤ 5 years	255(28.72)		122(40.94)		422(41.49)		463(47.63)	
6–12 years	444(50.00)		129(43.29)		443(43.56)		403(41.46)	
≥ 13 years	189(21.28)		47(15.77)		152(14.95)		106(10.91)	
**Physical activity- no. (%)**		0.009		0.042		< 0.001		0.006
Low	381(42.86)		135(45.30)		450(44.20)		423(43.47)	
Moderate	409(46.01)		140(46.98)		493(48.43)		460(47.28)	
Heavy	99(11.14)		23(7.72)		75(7.37)		90(9.25)	
**BMI- no. (%)**		< 0.001		< 0.001		< 0.001		0.038
< 25	120(13.53)		53(17.79)		167(16.42)		249(25.62)	
25–29.9	429(48.37)		123(41.28)		464(45.62)		403(41.46)	
≥ 30	338(38.11)		122(40.94)		386(37.95)		320(32.92)	
**WSI-no. (%)**		< 0.001		0.137		< 0.001		< 0.001
Low	213(23.99)		79(26.51)		274(26.94)		306(31.48)	
Low-middle	247(27.82)		90(30.20)		316(31.07)		276(28.40)	
Middle-high	328(36.94)		101(33.89)		372(36.58)		352(36.21)	
High	100(11.26)		28(9.40)		55(5.41)		38(3.91)	
**Alcohol consumption- no. (%)**		< 0.001		0.006		0.945		0.045
Yes	142(16.15)		43(14.78)		101(10.05)		114(11.83)	
No	737(83.85)		248(85.22)		904(89.95)		850(88.17)	
**Cigarette smoking -no. (%)**		0.311		0.441		0.049		< 0.001
Yes	213(24.23)		69(23.71)		232(23.08)		318(32.99)	
No	666(75.77)		222(76.29)		773(76.92)		646(67.01)	
**Opium consumption- no. (%)**		0.001		0.227		0.583		< 0.001
Yes	168(19.11)		60(20.62)		230(22.89)		307(31.85)	
No	711(80.89)		231(79.38)		775(77.11)		657(68.15)	
**Hypertension- no. (%)**		0.391		0.019		< 0.001		< 0.001
Yes	189(21.43)		83(28.23)		297(29.41)		317(32.82)	
No	693(78.57)		211(71.77)		713(70.59)		649(67.18)	
**Family history of diabetes in first-degree relatives- no (%)**		0.134		0.402		0.053		0.102
Yes	456(51.70)		152(51.70)		527(52.18)		452(46.79)	
No	426(48.30)		142(48.30)		483(47.82)		514(53.21)	
**Family history of diabetes in second-degree relatives- no (%)**		0.767		0.322		0.940		0.021
Yes	213(24.15)		65(22.11)		247(24.46)		208(21.53)	
No	669(75.85)		229(77.89)		763(75.54)		758(78.47)	
**Fatty liver- no. (%)**		< 0.001		< 0.001		< 0.001		0.409
Yes	167(18.93)		53(18.03)		165(16.34)		106(10.97)	
No	715(81.07)		241(81.97)		845(83.66)		860(89.03)	
**Hepatitis- no. (%)**		0.318		0.016		0.930		0.865
Yes	4(0.45)		3(1.02)		3(0.30)		3(0.31)	
No	878(99.55)		291(98.98)		1007(99.70)		963(99. 69)	
**Use of hepatotoxic drugs- no. (%)**		0.207		0.254		< 0.001		< 0.001
Yes	197(22.34)		79(26.87)		315(31.19)		304(31.47)	
No	685(77.66)		215(73.13)		695(68.81)		662(68.53)	
**Cholesterol- no. (%)**		< 0.001		0.003		< 0.001		< 0.001
Normal	394(44.32)		141(47.32)		433(42.53)		487(50.05)	
Elevated	495(55.68)		157(52.68)		585(57.47)		486(49.95)	
**Triglycerides- no. (%)**		< 0.001		0.005		< 0.001		< 0.001
Normal	557(62.65)		203(68.12)		596(58.55)		662(68.04)	
Elevated	332(37.35)		95(31.88)		422(41.45)		311(31.96)	
**LDL cholesterol- no. (%)**		< 0.001		< 0.001		< 0.001		0.001
Normal	633(71.28)		201(67.68)		699(68.73)		712(73.25)	
Elevated	255(28.72)		96(32.32)		318(31.27)		260(26.75)	
**HDL cholesterol- no. (%)**		0.855		0.479		0.679		0.594
Normal	885(99.55)		296(99.33)		1013(99.51)		970(99.69)	
Reduced	4(0.45)		2(0.67)		5(0.49)		3(0.31)	
**FBS- no. (%)**		< 0.001		< 0.001		< 0.001		< 0.001
Normal	677(76.15)		217(72.82)		696(68.37)		682(70.09)	
Elevated	212(23.85)		81(27.18)		322(31.63)		291(29.91)	

*Abbreviations:* *BMI* body mass index, *FBS* Fasting blood sugar, *AST* aspartate aminotransferase, *ALT*, alanine aminotransferase, *ALP* alkaline phosphatase, *GGT* γ-glutamyl transferase, *WSI* Wealth score index

Also, we calculated the predicted probabilities of diabetes and graphed them against the observed values of liver enzymes using bivariate logistic regression. There was a linear relation between elevated liver enzymes and probability of diabetes (Figure S1).

Table 3 gives the relation between the levels of liver enzymes with diabetes, using crude and four adjusted models. Elevated ALT, GGT, AST and ALP were related with diabetes in all crude and adjusted models (adjusted ORs: 1.81, 95%CI 1.51–2.17; 1.75, 95%CI 1.32–2.32; 1.77, 95%CI 1.50–2.08; 1.60, 95%CI 1.35–1.90 respectively).

**Table 3:** Odds ratios (95% confidence interval) for diabetes by the level of liver enzymes

**Characteristic**s	**Crude model**	**Adjusted model 1**	**Adjusted model 2**	**Adjusted model 3**	**Adjusted model 4**
	**OR (95%CI)**^**a**^	**OR(95%CI)**^**b**^	**OR (95%CI)**^**c**^	**OR(95%CI)**^**d**^	**OR(95%CI)**^**e**^
**ALT**					
Normal ALT	1	1	1	1	1
Elevated ALT	1.45(1.24–1.68)	2.19(1.85–2.60)	1.97(1.66–2.33)	1.96(1.64–2.33)	1.81(1.51–2.17)
**AST**					
Normal AST	1	1	1	1	1
Elevated AST	1.75(1.37–2.42)	1.92(1.47–2.51)	1.79(1.37–2.34)	1.78(1.35–2.35)	1.75(1.32–2.32)
**GGT**					
Normal GGT	1	1	1	1	1
Elevated GGT	2.22(1.94–2.56)	2.13(1.84–2.48)	1.98(1.71–2.31)	1.99(1.70–2.32)	1.77(1.50–2.08)
**ALP**					
Normal ALP	1	1	1	1	1
Elevated ALP	2.00(1.74–2.30)	1.65(1.42–1.92)	1.66(1.42–1.94)	1.67(1.42–1.96)	1.60(1.35–1.90)

^a^ The baseline model is stratified on the levels of liver enzymes

^b^ The adjusted model 1 is adjusted for confounding variables including age (continuous variable), gender (male/ female), education years (continuous variable) and wealth status index

^C^ The adjusted model 2 has additional adjustment for confounding variables related to lifestyle (cigarette smoking, alcohol drinking and opium consumption), body mass index (continuous variable) and physical activity level (continuous variable)

^d^ The adjusted model 3 has additional adjustment for hypertension (yes/no), family history of diabetes (first-degree relatives) (yes/no) and family history of diabetes (second-degree relatives) (yes/no)

^e^ The adjusted model 4 has additional adjustment for cholesterol (continuous variable), triglycerides (continuous variable), LDL cholesterol (continuous variable), HDL cholesterol (continuous variable), use of hepatotoxic drugs (yes/no) and fatty liver (yes/no)

The relation between the levels of liver enzymes within normal range with diabetes, using crude and four adjusted models are shown in Table 4. In subjects with normal levels of ALT, in all crude and adjusted models, a dose–response increase was seen with the highest ORs in the fourth quartile for diabetes. In model 4, in the subjects having the greater ALT, OR of diabetes in normal quartile 4 was 1.61(1.37–1.90). Within the normal range, the AST level was not positively associated with odds of diabetes. In subjects with normal levels of GGT, in all models, a dose–response increase was seen with the greatest ORs in the fourth quartile for diabetes. In adjusted model 4, in subjects having increased levels of GGT, the OR of diabetes in normal quartile 4 was 2.60 (2.17–3.11). In subjects with normal levels of ALP, in all models, the OR of diabetes in normal quartile 4 was significantly greater than the subjects in the first quartile (Table 4).

**Table 4:** Odds ratios (95% confidence interval) for diabetes by the level of liver enzymes within normal range

**Characteristic**s	**Crude model**	**Adjusted model 1**	**Adjusted model 2**	**Adjusted model 3**	**Adjusted model 4**
	**OR (95%CI)**^**a**^	**OR(95%CI)**^**b**^	**OR (95%CI)**^**c**^	**OR(95%CI)**^**d**^	**OR(95%CI)**^**e**^
**ALT**					
Normal Quartile 1	1	1	1	1	1
Normal Quartile 2	1.36(1.17–1.58)	1.36(1.16–1.60)	1.25(1.07–1.48)	1.26(1.06–1.49)	1.19(1.01–1.42)
Normal Quartile 3	1.90(1.65–2.18)	1.83(1.58–2.13)	1.64(1.40–1.91)	1.61(1.37–1.89)	1.42(1.20–1.67)
Normal Quartile 4	2.04(1.78–2.35)	2.25(1.93–2.61)	1.94(1.66–2.26)	1.88(1.60–2.21)	1.61(1.37–1.90)
Elevated ALT	2.19(1.83–2.62)	3.37(2.78–4.09)	2.86(2.34–3.50)	2.82(2.29–3.46)	2.40(1.94–2.97)
**AST**					
Normal Quartile 1	1	1	1	1	1
Normal Quartile 2	0.68(0.59–0.78)	0.62(0.54–0.72)	0.61(0.52–0.71)	0.61(0.52–0.71)	0.59(0.50–0.70)
Normal Quartile 3	0.81(0.71–0.91)	0.68(0.59–0.78)	0.66(0.57–0.76)	0.67(0.58–0.77)	0.63(0.54–0.73)
Normal Quartile 4	0.88(0.77–1.00)	0.82(0.71–0.94)	0.56(0.66–0.87)	0.75(0.65–0.87)	0.69(0.59–0.80)
Elevated AST	1.49(1.15–1.92)	1.51(1.14–2.00)	1.36(1.03–1.81)	1.36(1.02–1.82)	1.28(0.95–1.71)
**GGT**					
Normal Quartile 1	1	1	1	1	1
Normal Quartile 2	1.92(1.63–2.26)	1.79(1.51–2.12)	1.63(1.37–1.94)	1.61(1.35–1.93)	1.50(1.25–1.80)
Normal Quartile 3	2.40(2.04–2.81)	2.27(1.92–2.69)	1.98(1.67–2.35)	1.96(1.64–2.33)	1.75(1.46–2.10)
Normal Quartile 4	3.57(3.07–4.17)	3.63(3.08–4.27)	3.11(2.63–3.67)	2.99(2.52–3.55)	2.60(2.17–3.11)
Elevated GGT	4.63(3.89–5.53)	4.34(3.60–5.25)	3.72(3.06–4.50)	3.68(3.01–4.48)	3.10(2.51–3.82)
**ALP**					
Normal Quartile 1	1	1	1	1	1
Normal Quartile 2	1.17(1.01–1.37)	1.03(0.88–1.22)	0.99(0.85–1.17)	1.01(0.85–1.19)	0.98(0.83–1.17)
Normal Quartile 3	1.49(1.28–1.73)	1.20(1.02–1.41)	1.11(0.94–1.30)	1.13(0.96–1.33)	1.09(0.92–1.29)
Normal Quartile 4	1.97(1.71–2.28)	1.50(1.28–1.74)	1.40(1.20–1.64)	1.39(1.19–1.63)	1.30(1.10–1.54)
Elevated ALP	2.78(2.34–3.30)	1.96(1.63–2.36)	1.88(1.56–2.27)	1.90(1.56–2.31)	1.76(1.44–2.16)

^a^ The baseline model is stratified on the levels of liver enzymes

^b^ The adjusted model 1 is adjusted for confounding variables including age (continuous variable), gender (male/ female), education years (continuous variable) and wealth status index

^C^ The adjusted model 2 has additional adjustment for confounding variables related to lifestyle (cigarette smoking, alcohol drinking and opium consumption), body mass index (continuous variable) and physical activity level (continuous variable)

^d^ The adjusted model 3 has additional adjustment for hypertension (yes/no), family history of diabetes (first-degree relatives) (yes/no) and family history of diabetes (second-degree relatives) (yes/no)

^e^ The adjusted model 4 has additional adjustment for cholesterol (continuous variable), triglycerides (continuous variable), LDL cholesterol (continuous variable), HDL cholesterol (continuous variable), use of hepatotoxic drugs (yes/no) and fatty liver (yes/no)

In addition to adjustment for fatty liver, since some of the increased odds of diabetes probably are driven from residual confounding from fatty liver or its interaction effects with liver enzymes, we performed a sensitivity analysis in fatty liver and non-fatty liver subjects. However, adjusted model showed that elevated ALT, elevated AST, elevated GGT and elevated ALP in non-fatty liver subjects increased odds of diabetes (adjusted ORs: 1.80, 95%CI 1.46–2.20; 1.75, 95%CI 1.27–2.40; 1.76, 95%CI 1.47–2.10 and 1.62, 95%CI 1.36–1.94 respectively), and also elevated ALT and elevated GGT in fatty liver subjects increased the odds of diabetes (ORs: 1.78, 95%CI 1.19–2.67 and 1.96, 95%CI 1.32–2.92 respectively)( Table S1).

## Discussion

In the present cross-sectional study, we assessed the relation between serum liver enzymes and diabetes among participants of Rafsanjan Cohort Study. The frequency of elevated liver enzymes (ALP, AST, ALT and GGT) was significantly higher in diabetic subjects compared to non-diabetic subjects. Serum ALT, ALP and GGT levels had significantly positive relationship with age. In this investigation, the frequency of increased liver enzymes (ALP, AST & GGT) in the diabetic group has been greater in females relative to the males in accordance with a previous investigation [7]. Individual differences of body fat distribution and metabolism can account for gender differences.

Our findings showed a positive relation of higher serum concentrations of AST, ALT, GGT, and ALP with an increased odds of diabetes. Previous studies on relation of liver enzymes with the risk of diabetes were conducted in South Korea [23], Japan [24], Iran [11, 25], Western [2], China [26], Thailand [27] and India [13] with inconsistent findings. Some studies reported that liver enzymes improve diabetes prediction [28, 29], while others did not [25, 30]. One of the previous studies in the White and African-American populations revealed that ALT ≥ 26 IU/L substantially improved diabetes anticipation [28] and one of the Japanese studies detected the declined cut-off value for ALT (13 IU/L) [29]. Differences in reference values, age groups, individual habits as well as demography can partly or largely explain the differences. Ethnicity may also have an important role because in the separate analysis of Black and Hispanic participants, there was no considerable relation between hepatic markers and diabetes [31]. Moreover, a meta-analysis of 24 prospective investigations reported ORs of 1.34 (95% CI, 1.27 to 1.42) and 1.66 (95% CI, 1.31 to 2.09; 17 studies) in the highest quartile of GGT and ALT, respectively [32, 33]. Investigations with relatively small sample size that ranged between 36 and 208 diabetic subjects showed no relationship [34, 35], possibly due to lack of power.

A Mendelian randomization study presented underlying evidence of the relation of GGT to insulin resistance [36]. The majority of these investigations indicated the independent association between GGT and diabetes [24, 26, 37]. Another follow-up study from Korea revealed a strong dose–response association between the serum concentration of GGT and prevalence of diabetes [38]. Based on a number of studies, elevation of GGT is superior to ALT for the early detection of diabetes [39, 40]. Also, a cross-sectional research in Bangladeshi adults showed that the frequency of elevated ALP, AST, ALT and GGT was higher in diabetic individuals, but only elevated GGT showed independent relation with diabetes in that population [17]. A possible mechanism for the association between GGT level and risk of diabetes is the role of GGT in intracellular antioxidant defense systems in relation to the main function of modulating intracellular glutathione level [41]. Increasing oxidative stress would play a role in diabetes progression [42], and chronic oxidative stress results in reduced responsiveness to insulin and ultimately causes diabetes [43]. While the relevant mechanism has remained largely uncertain, the modifications of inflammation occurring due to oxidative stress are considered as one of the common steps in the pathogenesis of diabetes. In a number of studies, GGT and ALT have been both considerably related to the risk of diabetes [23, 25, 26, 37]. In contrast, a few studies reported the absence of remarkable relation between ALT and diabetes after adjusting a minimal or full array of risk factors for diabetes in the statistical models [39, 44].

Most previous studies analyzed AST, ALT and GGT levels in diabetic subjects, and only some investigations included ALP [7, 11, 17, 26]. The increased level of ALP in diabetic individuals in our study is in agreement with the earlier investigations in which ALP increased in diabetic people [11, 45, 46]. The Tehran Lipid and Glucose Study reported that increasing levels of GGT, ALP and ALT showed a positive association with diabetes. In that study, the only confounders included in models were gender, age, and BMI. In our study, more confounders were considered. The mechanism for relation of increased serum level of ALP with risk of diabetes remains unclear. A possible mechanism is that ALP showed a negative relation with adiponectin, a secreted hormone from adipose tissues [47]. Decreasing serum levels of adiponectin has known as a risk factor for progression to diabetes [48].

Although the precise mechanism of the relationship between increased serum levels of liver enzymes with the risk of diabetes remains unclear, the most likely explanation is NAFLD. Higher levels of the liver enzymes indicate the presence of NAFLD [49]. Many investigations indicated that NAFLD is strongly related to diabetes [50]. On the other hand, high free fatty acids in the liver may cause fasting hyperglycemia, hyperinsulinemia, and decreased insulin signaling, which eventually leads to diabetes [51, 52]. One of the other probable mechanisms linking liver enzymes to diabetes is liver inflammation [53] through increasing proinflammatory adipocytokines as well as decreasing anti-inflammatory adiponectin [54].

We also investigated the relationship between AST, ALT, GGT and ALP levels within their normal ranges with diabetes. Not only elevated AST, GGT, ALT and ALP levels were positively associated to diabetes, but also in individuals with normal serum levels of ALP, GGT and ALT, with raising enzyme concentration, the odds of diabetes increased with a dose–response relationship even after adjustment for all mentioned confounders. In agreement with the results of our study, some studies also showed that higher levels of ALT [11], GGT [55] and ALP [46] within normal range were associated with the risk of diabetes. Webber et al. showed that ALP was significantly associated with hypertension, cardiovascular disease, hypercholesterolemia, and diabetes after adjusting for gender, age, ethnicity and BMI. In comparison to the lowest quartile of ALP, the highest quartile had higher odds of heart disease, hypertension, hypercholesterolemia and diabetes [46]. In the Tehran Lipid and Glucose Study, increased levels of ALT within normal ranges were positively associated with risk of diabetes [11].

As far as we know, our study is the first study to investigate the association between GGT and ALP within normal ranges and diabetes in Iran. The association of elevated AST with the odds of diabetes was weaker than that of other enzymes, confirming previous studies [17, 55]. The stronger association for higher ALT than elevated AST is due to more prolonged plasma half-life of ALT or the greater specificity of ALT for liver injuries [56, 57].

The large sample size with extensive information about potential confounders was one of the main strengths of our research. Another major strength of our investigation was the adjustment for recognized diabetes risk factors such as demographic and lifestyle information, history of hypertension, family history of diabetes and BMI. Second, according to some of the former investigations, diabetes has been detected according to self-reports while in the present research, diabetes was diagnosed based on both self-reporting documents and measurement of FBS concentration. Nonetheless, there were a number of limitations in our research. First, the cross-sectional design of the study did not allow deriving any causal inferences. Accordingly, this relationship will be reconsidered in the follow-up phase of this prospective study. Secondly, hepatitis C and B infections were not screened. Diagnostic criteria with FBS alone (not in combination with 2hBG and HbA1c) that might lead to miscalculation of diabetes prevalence is another limitation of our study.

## Conclusions

Elevated levels of ALT, AST, GGT and ALP are related to a higher odds of diabetes. Also, increased serum levels of ALT, GGT and ALP even within normal range were independently related with the increased odds of diabetes. These results indicated the potential of elevated liver enzymes as biomarkers for the possible presence of diabetes.

## Supplementary Information

**Additional file 1:** **Figure S1.** Relationbetweenelevated liver enzymes and probability of diabetesusing bivariate logistic regression

**Additional file 2:** **Table S1.**Odds ratios (95% confidence interval) for diabetes by the level of liverenzymes in participants with and without fatty liver

## Data Availability

The datasets used during the current study are available on the Persian Adult Cohort Study Center, Rafsanjan University of Medical Sciences, Iran. The data is not available publicly. However, upon a reasonable request, the data can be obtained from the corresponding author.
