# Supplementary material for: Serum liver enzymes and diabetes from the Rafsanjan cohort study
Source: BMC Endocr Disord. 2022 May 12;22:127. doi: 10.1186/s12902-022-01042-2 (PMC9102258; doi:10.1186/s12902-022-01042-2)
Supplement: Supplementary file 1 — Additional file 1: Figure S1. Relationbetweenelevated liver enzymes and probability of diabetesusing bivariate logistic regression [file 12902_2022_1042_MOESM1_ESM.docx]

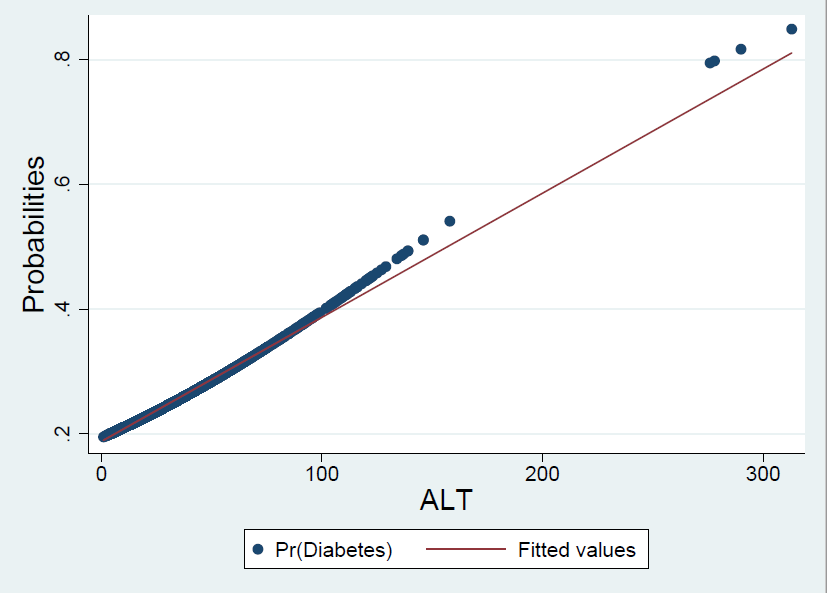

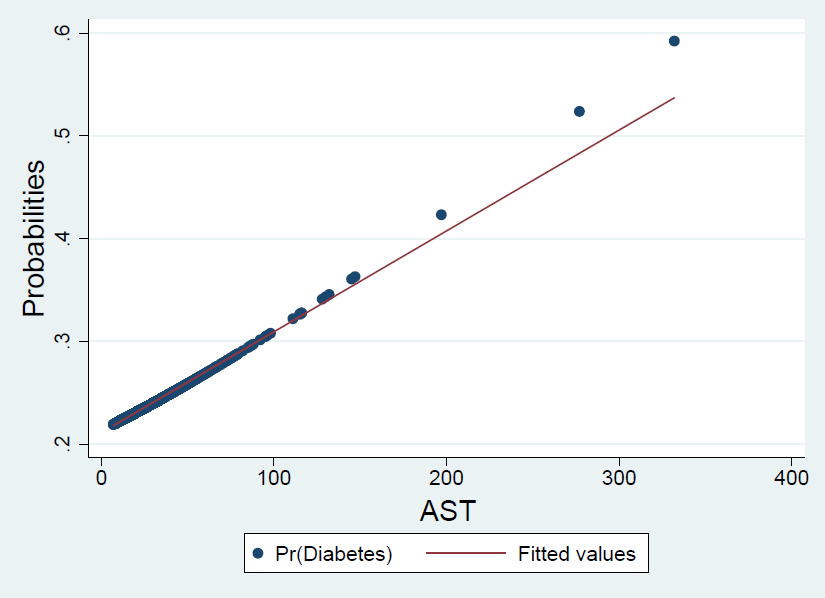

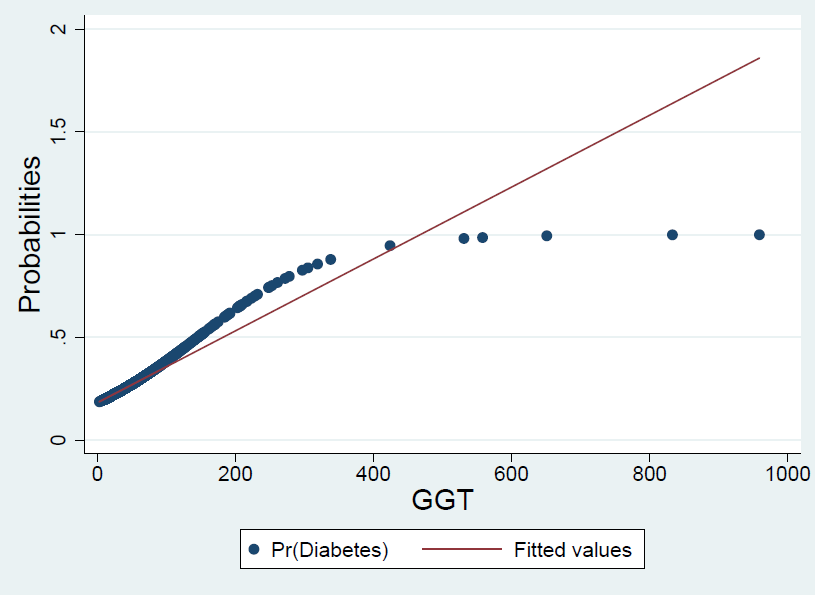

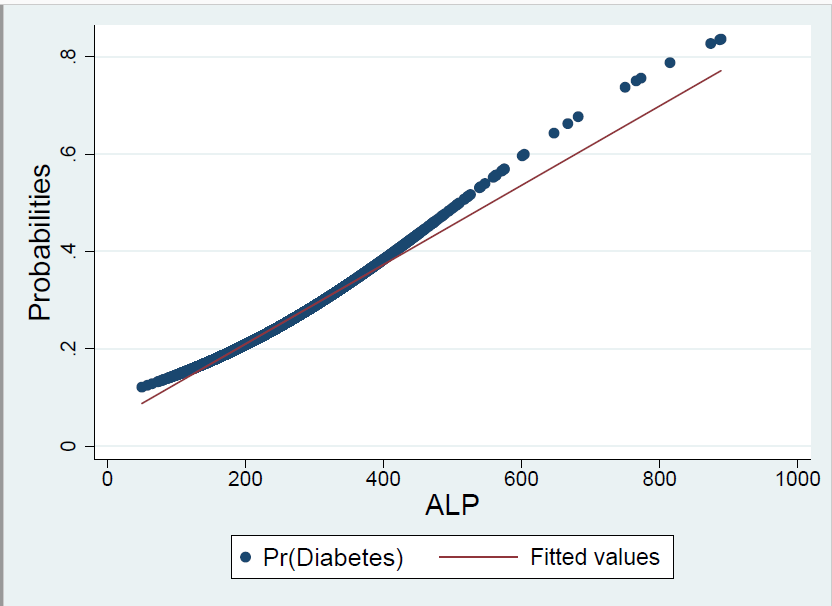


Figure S1. Relation between elevated liver enzymes and probability of diabetes using bivariate logistic regression
