# Supplementary material for: Serum liver enzymes and diabetes from the Rafsanjan cohort study
Source: BMC Endocr Disord. 2022 May 12;22:127. doi: 10.1186/s12902-022-01042-2 (PMC9102258; doi:10.1186/s12902-022-01042-2)
Supplement: Supplementary file 2 — Additional file 2: Table S1.Odds ratios (95% confidence interval) for diabetes by the level of liverenzymes in participants with and without fatty liver [file 12902_2022_1042_MOESM2_ESM.doc]

| **Table S1. Odds ratios (95% confidence interval) for diabetes by the level of liver enzymes in participants with and without fatty liver.** | | | | |
| --- | --- | --- | --- | --- |
| **Crude model** | **Non-fatty liver** | | **fatty liver** | |
|  | **Crude OR (95%CI)^a^** | **Adjusted OR(95%CI)^b^** | **Crude OR (95%CI)^a^** | **Adjusted OR(95%CI)^b^** |
| **ALT** | |  |  |  |
| Normal ALT | 1 | 1 | 1 | 1 |
| Elevated ALT | 1.39(1.17-1.66) | 1.80(1.46-2.20) | 1.23(0.87-1.73) | 1.78(1.19-2.67) |
| **AST** | |  |  |  |
| Normal AST | 1 | 1 | 1 | 1 |
| Elevated AST | 1.79(1.36-2.36) | 1.75(1.27-2.40) | 1.24(0.7-2.18) | 1.64(0.87-3.10) |
| **GGT** | |  |  |  |
| Normal GGT | 1 | 1 | 1 | 1 |
| Elevated GGT | 2.16(1.85-2.51) | 1.76(1.47-2.10) | 2.10(1.49-2.93) | 1.96(1.32-2.92) |
| **ALP** | |  |  |  |
| Normal ALP | 1 | 1 | 1 | 1 |
| Elevated ALP | 2.03(1.75-2.36) | 1.62(1.36-1.94) | 1.84(1.23 -2.77) | 1.54(0.96-2.47) |
| ^a^ The baseline model is stratified on the levels of liver enzymes.  ^b^ The adjusted model is adjusted for confounding variables including age (continuous variable), gender (male/ female), education years (continuous variable), wealth status index, confounding variables related to lifestyle (cigarette smoking, alcohol drinking and opium consumption), body mass index (continuous variable), physical activity level (continuous variable), hypertension (yes/no), family history of diabetes (first-degree relatives) (yes/no) and family history of diabetes (second-degree relatives) (yes/no), Triglycerides (continuous variable), LDL cholesterol (continuous variable), HDL cholesterol (continuous variable), use of hepatotoxic drugs (yes/no). | | | | |
